# The myxozoan parasite *Myxobolus bejeranoi* (Cnidaria: Myxozoa) infection dynamics and host specificity in hybrid tilapia aquaculture

**DOI:** 10.1017/S0031182023000240

**Published:** 2023-05

**Authors:** Keren Maor-Landaw, Itamar Avidor, Barbara Salti, Margarita Smirnov, Vera Brekhman, Tamar Lotan

**Affiliations:** 1 Marine Biology Department, The Leon H. Charney School of Marine Sciences, University of Haifa, Mt. Carmel, 3498838 Haifa, Israel; 2 Department of Fisheries and Aquaculture, Central Fish Health Laboratory, Ministry of Agriculture and Rural Development, 10803 Nir David, Israel

**Keywords:** Fishpond, host specificity, hybrid, infection, *in situ* hybridization, Myxozoa, parasite, quantitative PCR, tilapia

## Abstract

Nile × blue tilapia hybrid (*Oreochromis niloticus* × *O. aureus*) has become an important food fish in intensive freshwater aquaculture. Recently, the parasite *Myxobolus bejeranoi* (Cnidaria: Myxozoa) was found to infect hybrid tilapia gills at high prevalence, causing immune suppression and high mortality. Here, we explored additional characteristics of *M. bejeranoi*–tilapia interaction, which enable efficient proliferation of this parasite inside its specific host. Highly sensitive quantitative polymerase chain reaction (qPCR) and *in situ* hybridization analyses of fry collected from fertilization ponds provided evidence to an early-life infection of fish by a myxozoan parasite, occurring less than 3 weeks post-fertilization. Because *Myxobolus* species are highly host-specific, we next compared infection rates in hybrid tilapia and in both its parental species following a 1-week exposure to infectious pond water. Analysis by qPCR and histological sections showed that while blue tilapia was as susceptible to *M. bejeranoi* as the hybrid, Nile tilapia appeared to be resistant. This is the first report of differential susceptibility of a hybrid fish *vs* its parental purebreds to a myxozoan parasite. These findings advance our understanding of the relationship between *M. bejeranoi* and tilapia fish and raise important questions regarding the mechanisms that allow the parasite to distinguish between very closely related species and to infect a specific organ at very early-life stages.

## Graphical abstract

**Figure:**
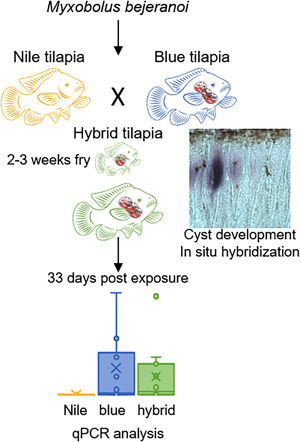

## Introduction

Tilapia (family Cichlidae) is the second most cultured fish worldwide (El-Sayed, 2020), and in Israel, it constitutes 60% of the total freshwater fish production (Hulata, 2014). Intensive aquaculture systems of earthen ponds containing an all-male hybrid of *Oreochromis niloticus* (Nile tilapia) females × *O. aureus* (Jordan/blue tilapia) males (Milstein *et al*., 2001; Eknath and Hulata, 2009; El-Sayed, 2020) have become a common practice all over the world and also in Israel (Milstein *et al*., 2001; Miao and Wang, 2020). This is because of the fast growth, tolerance to a wide range of environmental conditions and resistance to stress of the hybrid, as well as the ability of the parents to reproduce effectively in captivity (El-Sayed, 2020; Xiao *et al*., 2022). However, in the last 15 years, intense infections with a myxozoan parasite have been reported in Israeli fishponds.

Myxozoa is a large group of microscopic obligate endoparasites that has recently been placed within the phylum Cnidaria, alongside corals, sea anemones, jellyfish and hydroids [recently reviewed by Atkinson *et al*. (2018)]. They have complex life cycles, infecting both vertebrates (mostly fish) and invertebrates (mostly annelid worms) in freshwater and marine environments (Eszterbauer *et al*., 2015; Holzer *et al*., 2018). Transmission between hosts is achieved by 2 distinct types of waterborne spores: myxospores that develop in fish and when released infect the worm host, and actinospores that develop in worm and infect fish (Wolf and Markiw, 1984; Kent *et al*., 1994). An infected worm can propagate thousands of actinospores to the water column (Gilbert and Granath, 2001). Myxozoan parasites cause morbidity and mortality, such as whirling disease and proliferative kidney disease, in both farmed and wild fish populations worldwide (Okamura *et al*., 2015). Recently, the causative agent of hybrid tilapia infections, which occur at more than 50% prevalence during the summer season in commercial fish ponds (Maor-Landaw *et al*., 2022), was classified as *Myxobolus bejeranoi* (Lövy *et al*., 2018). The infection is limited to the gills, where cysts are formed on striated muscle of the gill filament base (Lövy *et al*., 2018). Nevertheless, we showed recently that the effects are systemic, as inhibition of the haematopoietic organs such as head, kidney and spleen results in immune suppression and high mortality (Maor-Landaw *et al*., 2022). Thus, *M. bejeranoi* infection of hybrid tilapia has high economic impact on commercial fish farms.

As all *Oreochromis* species, hybrid tilapia reproduce by maternal mouthbrooding. Females repeatedly release strings of 20–50 eggs to the bottom, which are then fertilized by the male as it passes over them. Immediately afterwards, the female returns and picks the fertilized eggs into its buccal cavity. Fertilized eggs will be incubated for 3–6 days in the female's mouth until hatching and brooding of the fry continues until the yolk sac is absorbed. Fry can swim freely in and out of the mother's mouth until the brooding period is completed in 1–2 weeks (Holden and Bruton, 1992; El-Sayed, 2020).

Here, we explore several characteristics of hybrid tilapia infection by *M. bejeranoi* in fish ponds in northeastern Israel. First, we examined if the water of the earthen ponds where fertilization takes place and the fry spend the first 3 weeks of their lives contain actinospores, and if so, whether the fry can be infected this early in their lifecycle. Another important aspect in parasite–host relationship is host specificity. Although in some cases multiple related species are infected, *Myxobolus* parasites are highly host-specific and plasmodia develop mostly in specific tissues or organs (Molnár and Eszterbauer, 2015; Forró and Eszterbauer, 2016). We were therefore interested to determine the specificity of *M. bejeranoi* to the hybrid tilapia by comparing infection rates to those of the parental purebred strains. To our knowledge, host specificity of hybrid fish *vs* its parental purebreds has not yet been studied in Myxozoa. Our study provides additional information on the capability of this myxozoan parasite in infecting its fish hosts.

## Materials and methods

### Study site

**Fig. 1.**
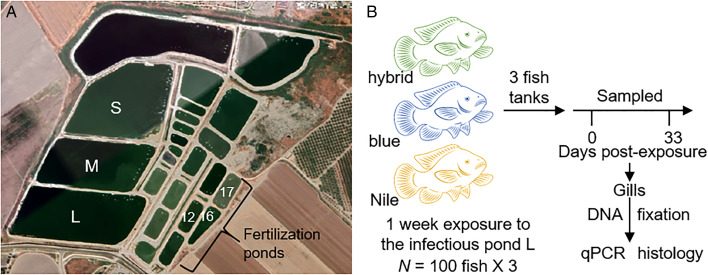
Layout of fish farms in Reshafim, Israel, and design of purebred experiments. (A) A Google Earth shot of the fish farms in Kibbutz Reshafim in northeastern Israel (32°28′53″N, 35°28′38″E). Pond L, where purebred experiments were conducted, and the fertilization ponds 12, 16 and 17, where early infection experiments were performed, are indicated. (B) Purebred experimental design. Approximately 100 fish from each tilapia strain, namely Nile tilapia (*Oreochromis niloticus*), blue tilapia (*O. aureus*) and hybrid tilapia (Nile × blue), were exposed to pond water using 3 confined cages of ~100 L (100 × 30 × 30 cm^3^). After 1 week of exposure, fish were translocated to the Central Fish Health Laboratory, Nir David, to 100 L indoor aquarium tanks. Fish were sampled before exposure and at 0 and 33 days post-exposure (dpe). Gill tissue was sampled for DNA extraction and qPCR analysis and was fixed for histology.

Research was carried out in earthen fishponds in the fish farms of Kibbutz Reshafim in northeastern Israel (32°28′53″N, 35°28′38″E; Fig. 1A). The rearing ponds (L, M, S in Fig. 1A) are 3 m deep and between 40 000 and 60 000 m^2^ in size. The ponds are supplied with water from the Hasi River through a circulation system and water salinity is about 1 ppt. At the bottom of the ponds, the thick sediment comprised a 4–5 cm layer of small-sized stones (0.5–1.5 cm in diameter) and then condensed mud. Fertilization ponds (12, 14, 16 in Fig. 1A) are situated in the same compound. Their characteristics are similar to those of the rearing ponds, except for their smaller size of approximately 5000 m^2^. Hybrid tilapia (Nile × blue tilapia) are cultured in the rearing ponds. The genetic diversity of the hybrids is unknown, because a variety of hybrids have been bred by the local aquaculture industry for many generations. Fish fertilization is carried out in 2–3 cycles during early summer by introducing males and females to the fertilization ponds. After 3 weeks, the fry are moved to a concrete pool, where they undergo hormonal sex reversal treatment using methyl testosterone for 3 weeks (Mojekwu, 2014). Then, they are usually transferred to intermediate pools and after about 1 month, juvenile fish are placed in the rearing pond.

### Detection of waterborne actinospores

#### Water collection and filtration

Water samples were taken from fertilization pond 12 on 30 June and 13 July 2021. Water samples were filtered first through a 60 *μ*m pore-size nitrocellulose membrane (Merck Millipore Ltd, NY6004700, MA, USA) and then through an 11 *μ*m membrane (Merck Millipore Ltd, NY1104700, MA, USA). The first membrane filtered out much of the larger plankton and detritus, whereas the pore size of the second membrane was appropriate for trapping *M. bejeranoi* actinospores. The 11 *μ*m membrane was collected after filtering 1–2 L of water, as 1 replicate, and was stored at −20°C for DNA extraction and polymerase chain reaction (PCR).

#### DNA extraction and PCR

Filter membrane was incubated with 1 mL of lysis buffer [40 mm ethylenediaminetetraacetic acid (EDTA), 50 mm Tris HCl (pH 8.3) and 0.75 M sucrose], 10 mg mL^−1^ of proteinase K and 10% sodium dodecyl sulfate (SDS) for 1 h at 55°C with occasional vortexing, as previously described (Massana *et al*., 1997), with minor modifications. Following phenol–chloroform–isoamyl alcohol phase separation steps, 1/10 volume of 3 M sodium acetate (pH 5.2) was added along with 2 volumes of ethanol. Following incubation for 20 min at −80°C and 15 min centrifugation at 4°C, DNA pellet was washed with 80% ethanol, and resuspended in PCR-grade water. PCR for detecting *M. bejeranoi* DNA was conducted using primers for *M. bejeranoi* small subunit ribosomal RNA gene (SSU rRNA; NCBI accession number MF401455; Mb 18S F186, Mb 18S R849) (Table S1) (Lövy *et al*., 2018). As a positive control, universal eukaryotic primers for 18S (Hadziavdic *et al*., 2014) were amplified. PCR was performed in 25 *μ*L volumes with 0.02 unit *μ*L^−1^ of Phusion Hot Start Flex DNA Polymerase (Biolabs), 1× of Phusion HF 5× Buffer, 200 *μ*M of deoxynucleoside triphosphates (dNTPs), 0.5 *μ*M of forward and reverse primers and 1 *μ*L (100–250 ng) of template. Denaturation of DNA (98°C for 5 min) was followed by 35 cycles of amplification (98°C for 10 s, 67°C for 10 s and 72°C for 30 s), and was ended by a 5 min extension (72°C). PCR products were run in a 1% agarose gel and the presence of actinospores was confirmed by a positive result for *M. bejeranoi* primers.

### Early infection experiments

#### Collection of fish samples

Females and males were introduced to fertilization ponds 16 and 12 on 25 May and 14 June 2021, respectively (Table 1). On 14 June, about 3 weeks after the introduction of parents, fry were collected from pond 16 and then translocated to 100 L indoor aquarium tanks in the Central Fish Health Laboratory, Nir David. Samples were collected on 20 June and then again on 30 June. Fry from pond 12 were extracted from females’ mouths into buckets with clean water, and then translocated to aquarium tanks. Samples were collected on the day of collection on 30 June and then on 13 July and 23 August (Table 1). Additional samples from fertilization ponds were collected during summer 2022. Females and males were introduced to fertilization ponds 17 and 16 on 25 and 31 May 2022, respectively. On 14 June, fry were collected from pond 17, both from females’ mouths and from the pond water (0.008 g). On 22 June, pond 16 was sampled; and on 10 July, fry were collected from ponds 12 and 17 as a mixture (Table 1). Samples were treated with TRIzol reagent (ThermoFisher Scientific) and kept at −80°C or were fixed for *in situ* hybridization (ISH).

**Table 1.** Summary of the results of early-life Myxozoa infection experiments during 2021 and 2022 seasons

Date parents introduced to the pond	Fertilization pond no.	Sampled source	Date of transfer to clean tank	Sampling date	*N*	Infection %	Actinospores identified
25/05/2021	16	Pond water	14/06/2021	20/06/2021	10	70	
				30/06/2021	10	80	
14/06/2021	12	Females mouths	30/06/2021	30/06/2021	30	0	v
				13/07/2021	10	0	v
				23/08/2021	11	0	
25/05/2022	17	Pond water	–	14/06/2022	12	33.3	
25/05/2022	17	Females mouths	14/06/2022	14/06/2022	12	0	
31/05/2022	16	Pond water	–	22/06/2022	12	58.3	
14, 20/06/2022	12 + 17	Pond water	–	10/07/2022	12	91.7	

#### DNA extraction

DNA extraction was conducted using TRIzol reagent (ThermoFisher Scientific) according to the manufacturer's instructions, with minor modifications. To a 0.1 g of frozen sample, 1.5 mL of TRIzol was added and the tissue was homogenized using 4 3 mm glass beads (CS Chemicals Ltd) and a TissueLyser II (Qiagen) for 3 min at 30 Hz. Following incubation with chloroform (Sigma-Aldrich) and centrifugation for 15 min at 12 000 ***g***, 4°C, the upper aqueous phase containing RNA was discarded, while the interphase and organic phases were utilized for DNA extraction. DNA quantification and purity were assessed using Nanodrop 2000c spectrophotometer (ThermoScientific).

#### Evaluation of infection severity by quantitative PCR

To detect low levels of *M. bejeranoi* DNA in fish gills, quantitative PCR (qPCR) was performed. Specific qPCR primers were utilized to amplify *M. bejeranoi* SSU rRNA gene (NCBI accession number MF401455) (Lövy *et al*., 2018) and tilapia *β*-actin gene was used as a normalizer gene (Zhou *et al*., 2019) (Table S1) for the comparative ΔΔCTs method. qPCR was performed on 25 ng DNA as previously described (Maor-Landaw *et al*., 2022) and normalized expression levels were calculated.

#### Whole-mount *in situ* hybridization

For whole-mount ISH, an amplicon of 663 bp generated using *M. bejeranoi* SSU rRNA gene (primers Mb 18S F186, Mb 18S R849; Table S1) was cloned into pJET1.2/blunt cloning vector (ThermoFisher Scientific) and then transformed into competent bacteria by heat shock of 42°C for 1 min, followed by 4°C for 2 min. Bacteria were grown in liquid LB at 37°C overnight and plasmids were extracted using HiSpeed® Plasmid Midi Kit (Qiagen). A riboprobe was constructed by transcription of PCR products, with T7 promoter linked to the 3′ end of the amplicon, using MEGAscript kit (Ambion) and DIG RNA Labeling Mix kit (Roche). The product of *in vitro* transcription was treated with DNase and then purified and cleaned using NucleoSpin RNA cleanup (Macherey-Nagel).

ISH was performed on samples from fertilization ponds of 3–4-week-old fry collected on 10 July 2022, fry collected from females’ mouths on 14 June 2022 and 10-week-old fry collected from pond S on 3 August 2022. A gill sample of about a 3-month-old severely infected fish with developed cysts was obtained in August 2020 and preserved in 100% ethanol at −20°C following 24 h fixation with 4% paraformaldehyde.

Whole-mount ISH was performed following a previously reported protocol (Thisse and Thisse, 2008), with some modifications. Whole fry were fixed in 4% paraformaldehyde for 24 h, followed by removal of pigmentation with hydrogen peroxide. Permeabilization was achieved by incubating with proteinase K (10 *μ*g mL^−1^) for 30 min, and hybridization was accomplished in 60°C for 2–6 days with 1 *μ*g mL^−1^ of probe (anti-sense and sense as a negative control). Anti-DIG antibody was diluted at 1/4000 with blocking solution.

### Evaluating the infectious potential of the fish pond water

To assess the level of actinospores in the pond water, throughout the summer of 2021, naïve fish were placed in pond L in ~100 L cages (100 × 30 × 30 cm^3^) that were tailor-made of a PVC frame coated with a 5 mm sized mesh, allowing for free water flow from the pond. Fish were exposed to pond water for 1 day, and then euthanized with 1 mL L^−1^ of 2-phenoxyethanol (Sigma-Aldrich). Gills were sampled into TRIzol reagent (ThermoFisher Scientific), DNA was extracted and qPCR for *M. bejeranoi* SSU rRNA gene was conducted as detailed above. Water temperatures were recorded constantly using a temperature data logger (HOBO), and on August 2021, mean temperature was 30.25°C (s.d. ± 0.68).

### Purebred experiments

#### Experimental layout

To assess host specificity of *M. bejeranoi* in infecting the hybrid, the 2 parental purebred tilapia species, along with the hybrid fish, were exposed to infectious pond water using 3 confined cages (as described above) for a 1-week period in August 2021. Each cage contained ~100 fish of the species blue tilapia (hatched on 8 June 2021; mean weight 3.04 g), Nile tilapia (hatched on 21 May; mean weight 2.94 g) or Nile × blue tilapia hybrid (hatched on 31 June; mean weight 2.98 g). Before exposure, 5 representative fish of each species were screened and found negative for pathogens. After exposure, fish were translocated to 100 L indoor aquarium tanks in the Central Fish Health Laboratory (Fig. 1B). Tanks had a flow-through system with dechlorinated tap water at a temperature of ~25°C. Fish were fed daily with commercial pellets.

#### Collection of fish samples, DNA extraction and qPCR

Experimental exposed fish were sampled at 0 and 33 days post-exposure (dpe) (10 < *n* < 30). Unexposed control fish were collected before the beginning of the experiment (11 < *n* < 15). Fish gills were sampled as described above. DNA was extracted using TRIzol reagent and followed by qPCR, as described above.

#### Histology analysis

Gills dissected from hybrid, Nile and blue tilapia were fixed in 10% neutral-buffered formalin, dehydrated in a graded ethanol series and embedded in 2-hydroxyethyl methacrylate. Then, 3 *μ*m-thick sections were generated using a Leica RM 2245 microtome. Sections were stained with haematoxylin and eosin according to standard protocol.

#### Statistical analysis

Normal distribution was tested by Shapiro–Wilk test and data that were not normally distributed were tested for significance using Mann–Whitney non-parametric test (SPSS software v. 27). Statistical significance was defined as *P* < 0.05.

## Results

To determine if hybrid tilapia at early-life stages can be infected by *M. bejerano*i, we examined fish fry of different lifecycle stages from fertilization and rearing ponds in commercial fish farms of Kibbutz Reshafim (Fig. 1A) during 2 consecutive summers. We collected fry that have completed metamorphosis and brooding period and swam freely in the pond water, as well as fry that were extracted from female mouths, at post-larva stage some still bearing their yolk sac to some extent (Ismarica *et al*., 2022).

In the first season, the summer of 2021, fry that had been collected from pond 16 were analysed by qPCR 6 and 16 days later (20 and 30 June; Table 1). Results showed 70 and 80% of infection in the first and second sampling points, respectively (Table 1). By contrast, in the fry of mixed developmental stages collected from 4 females’ mouths 2 weeks after introducing males and females to fertilization pond 12 (30 June), no infection was detected (Table 1). To corroborate this result, additional individuals were kept in aquarium tanks in clean flow water system and were re-examined twice, after 2 weeks and after a month, with no indication for infection. Notably, PCR analysis of water collected from fertilization pond 12 on both sampling occasions (30 June and 13 July) showed the presence of waterborne actinospores (Table 1).

In the second season, during early summer 2022, we sought to validate the infection of hybrid tilapia fry by *M. bejeranoi* shown in the 2021 trials. We found in 3 different fertilization cycles that indeed fry which are less than 3 weeks old are infected with the parasite and that the percentage of infection increased during the season (Table 1). Additionally, we repeated the analysis of fry at early developmental stages extracted directly from females’ mouths (on 14 June) 3 weeks after introducing males and females to fertilization pond 17 (Table 1). Results showed that while 33% of the fry sampled from the water of the same pond at the same time point were infected, brooding fry were not.

**Fig. 2.**
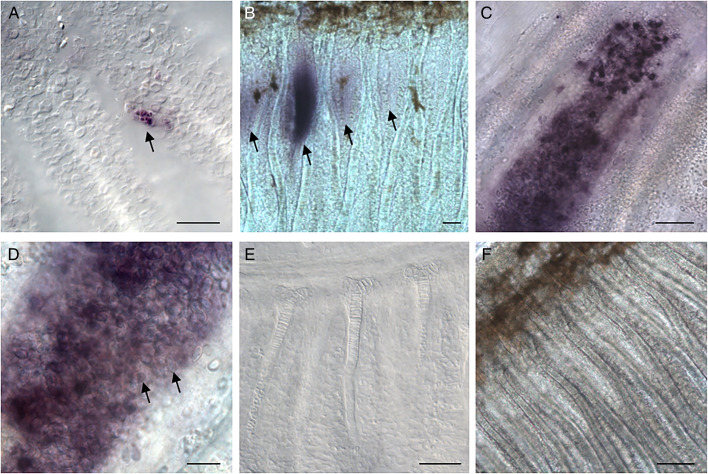
Whole-mount *in situ* hybridization for *M. bejeranoi* SSU rRNA shows infection progression. (A) Early infection of 3–4-week-old fry. Bar, 50 *μ*m. (B) Cysts at different developmental stages (mark with arrows) in 6-week-old fry. Bar, 100 *μ*m. (C) A cyst full of spores at an advanced infection stage in ~3-month-old juveniles. Bar, 50 *μ*m. (D) A close up of mature cysts with spores (mark with arrows). Bar, 20 *μ*m. (E) Sense control in 6-week-old fry. Bar, 50 *μ*m. (F) Non-infected control fry collected from females’ mouths. Bar, 100 *μ*m.

To visualize the early onset of infection, we performed whole-mount ISH with a specific probe targeting *M. bejeranoi* SSU rRNA gene. Results showed specific staining in the gills of 3–4-week-old infected fry (Fig. 2A). The analysis of older fry at 10 weeks of age revealed developing cysts (Fig. 2B), which later produced myxospores (Fig. 2C and D). No staining was apparent in control samples hybridized with the sense probe (Fig. 2E) or in fry collected from female's mouths hybridized with the anti-sense probe (Fig. 2F).

**Fig. 3.**
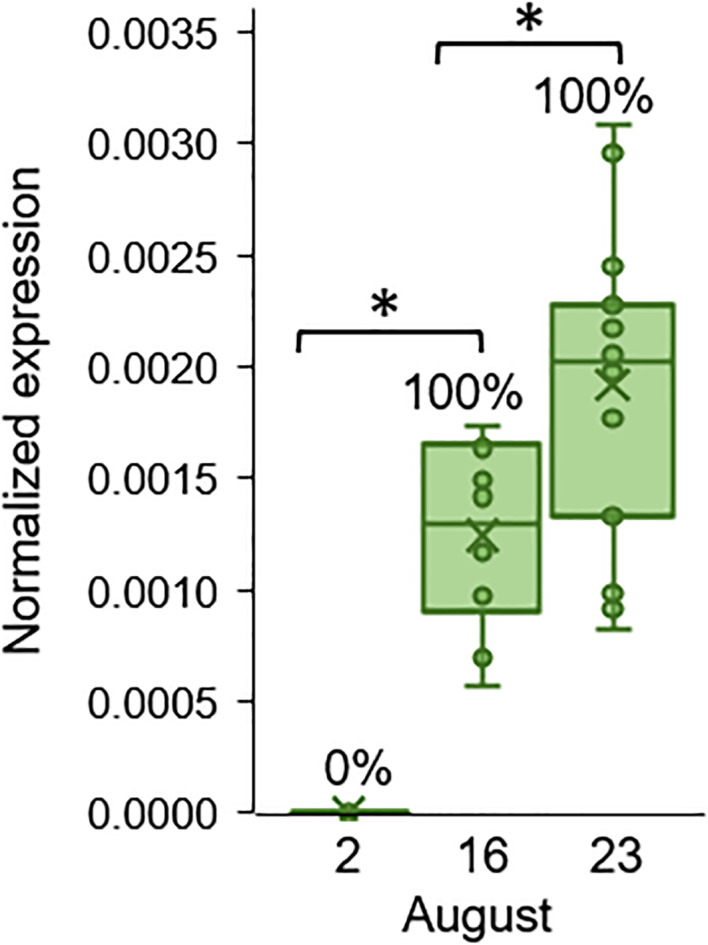
Normalized infection severity of hybrid tilapia in pond L during August 2021 following a 24 h exposure to pond water (9 < *n* < 15). Asterisks indicate statistically significant differences between the groups (*P* < 0.05 Mann–Whitney test). Percentages of infected fish are indicated above the bars.

These results indicate that the water of the fertilization ponds, in which new generations of cultured hybrid tilapia are hatched, contain *M. bejeranoi* actinospores that may infect young fry. We therefore proceeded to evaluate infection dynamics in the rearing ponds using fish that had been extracted from females’ mouths in fertilization pond 12 (on 14 June 2021), which were clear of myxozoan infection. Those naïve juveniles (0.24 g) were exposed to the water of pond L at 3 time points in August 2021 for 24 h, using cages. As determined by qPCR (Fig. 3), relative infection severity increased gradually demonstrating significant change (*P* < 0.05 Mann–Whitney test). Additionally, on July 13, actinospores were detected in the pond water.

**Fig. 4.**
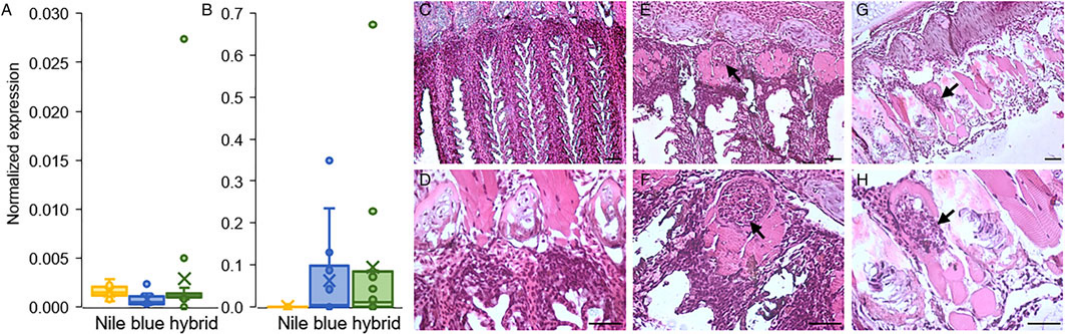
Progression of *M. bejeranoi* infection in 3 tilapia species. (A, B) Normalized qPCR analysis results for expression of *M. bejeranoi* SSU rRNA in Nile, blue and hybrid tilapia at 0 dpe (A) and 33 dpe (B) after a 1-week exposure to the pond water (11 < *n* < 15 for each fish species and time point). At 0 dpe, all 3 fish species displayed low levels of infection. At 33 dpe, there is a significant increase of infection level (*P* < 0.05 Mann–Whitney test) in either blue or hybrid compared to Nile tilapia. In uninfected control fish, infection levels were 0 (data not shown). The results are normalized to tilapia *β*-actin. (C–H) Histological sections show cyst-free tissue in Nile tilapia (C, D) and cyst development (marked with arrow) in blue tilapia (E, F) and hybrid tilapia (G, H). Bars in C, E and G are 50 *μ*m and in D, F and H 20 *μ*m.

Having established that the water of pond L is infectious (Fig. 3), we exposed the 2 parental purebred species, Nile and blue tilapia, alongside the hybrid tilapia, to the pond water for 1 week. The analysis of exposed fish at the end of the week revealed minor parasitic load in all 3 species (Fig. 4A). However, 33 days later, differences in infection patterns were observed. Whereas infection severity in blue and hybrid tilapia was similarly high, in Nile tilapia, copy numbers remained very low. Both blue and hybrid tilapia infection states after 33 days differed significantly from those of Nile tilapia (*P* < 0.05 Mann–Whitney test) (Fig. 4B). These differences in susceptibility to *M. bejeranoi* infection were further verified by histological analysis, which showed plasmodia formation in the gills of blue and hybrid tilapia, but not of Nile tilapia (Fig. 4).

## Discussion

In this study, we addressed several gaps in knowledge in the relationship between *M. bejeranoi* and cultured tilapia fish. In Israel, intensive aquaculture systems contain several pond types and fish are transferred from one pond to another. Thus, characterizing infection dynamics in the various ponds is important for a better understanding of the infection mechanism, as well as for planning and operating these facilities.

Infection rates in the fish pond labelled L peaked during August, as we observed in pond M in our previous study (Maor-Landaw *et al*., 2022). This result is not surprising, as many studies have linked myxozoan infection with seasonal increase in temperatures (Schmidt-Posthaus and Wahli, 2015). *Myxobolus bejeranoi* actinospores were found not only in the larger rearing ponds, but also in the fertilization ponds, where new offspring are generated in several cycles each season and where the sediment layout is similar. Furthermore, here we show that *M. bejeranoi* actinospores are capable of infecting hybrid tilapia fry. Given that the hatching and brooding period lasts 1–2 weeks (El-Sayed, 2020), the collected fry were only 3 weeks of age or younger. Ontogeny studies have shown that both blue and Nile tilapia complete metamorphosis in 15 days, when the yolk sac is absorbed (Holden and Bruton, 1992; Fishelson and Bresler, 2002). Thus, the fry that were infected by *M. bejeranoi* should have already developed gills, which are targeted by the parasite. Prior to their release from the female's mouth, brooding fry have the opportunity to swim out into the water (Lowe, 1955). Thus, fry obtained from females’ mouths were exposed to *M. bejeranoi* actinospores, but they were not infected. This could be due to underdeveloped gill lamellae (Holden and Bruton, 1992), or because of less exposure to the pond water.

There are several indications for myxozoan infection of fish larvae. The earliest reported infection that we found is of fry at about 1 month of age, which were infected by *Thelohanellus wuhanensis* (Wang *et al*., 2001). *Myxobolus cerebralis*, which is associated with the destruction of cartilage in juvenile salmonids, can infect fish at 7 weeks post-hatching (Ryce *et al*., 2005; Eszterbauer *et al*., 2015). Additional myxozoan species that infect juvenile fish are *Tetracapsuloides bryosalmonae*, *Henneguya ictaluri* (Fontes *et al*., 2015), *Thelohanellus wangi* (Yuan *et al*., 2015) and *Gadimyxa atlantica* (Holzer *et al*., 2010).

Fish at early-life stages are protected by a combination of passive immunity, transmitted from maternal sources, and innate immunity components, that include immunoglobulins, complement factors, lysozymes, protease inhibitors, lectins and serine proteases-like molecules (Swain and Nayak, 2009). This transition phase, after which the defence system becomes more adaptive-governed and eventually a fully responsive immunity, may last several weeks, depending on species and environmental conditions (Zapata *et al*., 2006). These circumstances render the fry more vulnerable to pathogens. Moreover, the direct contact of the gills with the pond water when the immune system is yet to be fully developed and responsive may facilitate the entry invasion of parasites such as *M. bejeranoi* (Vadstein *et al*., 2013). Our results indicate that when juveniles are transferred into the larger ponds, where most of their growth occurs, they may already be infected with the parasite. In the large rearing pond, where the fish spend several months, more fish–actinospore encounters are likely to happen, which would result in increased parasite load in the fish's gills. Ultimately, an immune-compromised individual will continuously be challenged by various opportunistic pathogens (Maor-Landaw *et al*., 2022).

Hybrid tilapia are the second most cultured strain of tilapia in the world and are the main strain in Israel (Miao and Wang, 2020). The natural distribution of Nile and blue tilapia largely overlaps in Africa and the Middle East, but hybridization was never documented in the wild (Rognon and Guyomard, 2003). However, given the high host specificity of *Myxobolus* species (Molnár and Eszterbauer, 2015), we investigated infection patterns in the 2 parental species, Nile and blue tilapia. Fish hybridization through interspecific breeding is exploited in aquaculture to produce offspring with improved traits (Hulata, 2001). Hybrid tilapia of Nile females and blue males is characterized by greater tolerance to cold and salinity (Hulata, 2001), improved growth performance and haematological metabolic indices and higher survival rates (Xiao *et al*., 2022). The hybrid growth vigour is supported by higher expression of growth hormone and insulin-like growth factor 1, compared to its parental species (Zhong *et al*., 2019; Zhou *et al*., 2019).

Hybrid tilapia is an example for heterosis, which is hybridization between different strains that produces an offspring with a superior phenotype and fitness (Chen, 2013). Heterosis has played a major role in shaping genetic variation that can be recombined and sorted into many new species when presented with the ecological opportunity, as was shown for haplochromine cichlid fishes of Lake Victoria (Meier *et al*., 2017). Thus, hybridization can also generate new habitats and niches for parasites (Krasnovyd *et al*., 2017). For example, cyprinid hybrids were shown to harbour more parasite species as compared to their parental species. However, parasite abundance was lower (Šimková *et al*., 2013; Krasnovyd *et al*., 2017), supporting an advantage of these heterotic hybrids in immune resilience (Cnaani *et al*., 2004; Šimková *et al*., 2015; Griffin *et al*., 2020; Šimkova *et al*., 2021).

Nonetheless, hybrids were documented to display susceptibility to different parasitic species (Krasnovyd *et al*., 2017). Indeed, when hybrid tilapia was exposed to *M. bejeranoi*, it exhibited high susceptibility and a progressed infection after 1 month. Our results show that the susceptibility of hybrid tilapia and its parental species Nile and blue tilapia to *M. bejeranoi* is of a non-additive nature, meaning the hybrid is not an intermediate between the parental species (Chen, 2013). These results are in accordance with the dominance scenario, which was coined by Fritz *et al*. to describe a hybrid whose susceptibility is similar to that of one of its parental taxa (Fritz *et al*., 1994).

Recently, we have demonstrated in a transcriptomic analysis that *M. bejeranoi* represses the immune system of its hybrid tilapia host by downregulating key immune-related genes, allowing for its efficient proliferation (Maor-Landaw *et al*., 2022). Whether blue tilapia infected with *M. bejeranoi* also undergoes immune suppression is yet to be tested by compared gene-expression patterns with the resilient Nile tilapia. Thus, the genetic mechanism underlying the resilience pattern (Fritz *et al*., 2003) presented here is unclear and was not within the scope of this study. Future studies should deploy omics tools along with epigenetics approaches and comprehensive quantitative trait locus-based phenotyping (Lippman and Zamir, 2006; Chen, 2013; Shen *et al*., 2017; Xiao *et al*., 2022) to uncover possible allelic interactions between parental genomes and altered programming of genes, which would shed light on the molecular apparatus that governs this phenomenon.

In summary, in this study, we show a novel early-life infection of hybrid tilapia with *M. bejeranoi* occurring already less than 3 weeks after fertilization, while fry are still in the fertilization ponds. Additionally, *M. bejeranoi* can also infect one of the hybrid's parental species, blue tilapia, with high prevalence that is similar to that of the hybrid. These findings open new questions regarding the mechanism allowing the parasite to distinguish the suitable host from a closely related species, and be organ-specific at very early-life stages.

## Data Availability

The authors confirm that the data supporting the findings of this study are available within the article. Raw data are available from the corresponding and first author.

## Supplementary material

For supplementary material accompanying this paper visit https://doi.org/10.1017/S0031182023000240. click here to view supplementary material
