# Supplementary material for: The myxozoan parasite Myxobolus bejeranoi (Cnidaria: Myxozoa) infection dynamics and host specificity in hybrid tilapia aquaculture
Source: Parasitology. 2023 Mar 10;150(6):524–30. doi: 10.1017/S0031182023000240 (PMC10260292; doi:10.1017/S0031182023000240)
Supplement: Supplementary file 1 [file S0031182023000240sup001.pdf]

Table S1 - Primers used in this study for PCR, ISH and qPCR.

PCR/ISH

**Primer**	**Sequence**	**Reference**
Myxobolus bejeranoi SSU rDNA - F186	CCTTTGGCTACCAGCTTGGA	Maor-Landaw et al. 2022
Myxobolus bejeranoi SSU rDNA - R849	ACCAAGCTGATCCCGAGTTG	
universal 18S SSU rRNA - F566	CAG CAG CCG CGG TAA TTC C	Hadziavdic et al. 2014
universal 18S SSU rRNA - R1200	CCCGTGTTGAGTCAAATTAAG C	

qPCR

**Primer**	**Sequence**	**Reference**
Myxobolus bejeranoi SSU rDNA - F153	TAGGAGGTGGTGAAGAGAA	Maor-Landaw et al. 2022
Myxobolus bejeranoi SSU rDNA - R235	GACTTGCCCTCCATTAGTT	
hybrid tilapia β-actin - F1069	GCCCCACCTGAGCGTAAATA	Maor-Landaw et al. 2022
hybrid tilapia β-actin - R1168	CATCGTACTCCTGCTTGCTGAT	
